# New Function for an Old Enzyme: NEP Deficient Mice Develop Late-Onset Obesity

**DOI:** 10.1371/journal.pone.0012793

**Published:** 2010-09-16

**Authors:** Matthias Becker, Wolf-Eberhard Siems, Reinhart Kluge, Florian Gembardt, Heinz-Peter Schultheiss, Michael Schirner, Thomas Walther

**Affiliations:** 1 Department for Biochemical Neurobiology, Leibniz-Institut für Molekulare Pharmakologie, Berlin, Germany; 2 Max-Rubner-Laboratorium, Deutsches Institut für Ernährungsforschung, Potsdam-Rehbrücke, Germany; 3 Centre for Biomedical Research, Hull York Medical School, University of Hull, Hull, United Kingdom; 4 Department for Experimental Cardiology, Excellence Cluster Cardio-Pulmonary System, Justus-Liebig-Universität Giessen, Giessen, Germany; 5 Department for Cardiology, Charité-Universitätsmedizin, Berlin, Germany; 6 Mivenion GmbH, Berlin, Germany; University of Minnesota, United States of America

## Abstract

**Background:**

According to the World Health Organization (WHO) there is a pandemic of obesity with approximately 300 million people being obese. Typically, human obesity has a polygenetic causation. Neutral endopeptidase (NEP), also known as neprilysin, is considered to be one of the key enzymes in the metabolism of many active peptide hormones.

**Methodology/Principal Findings:**

An incidental observation in NEP-deficient mice was a late-onset excessive gain in body weight exclusively from a ubiquitous accumulation of fat tissue. In accord with polygenetic human obesity, mice were characterized by deregulation of lipid metabolism, higher blood glucose levels, with impaired glucose tolerance. The key role of NEP in determining body mass was confirmed by the use of the NEP inhibitor candoxatril in wild-type mice that increased body weight due to increased food intake. This is a peripheral and not a central NEP action on the switch for appetite control, since candoxatril cannot cross the blood-brain barrier. Furthermore, we demonstrated that inhibition of NEP in mice with cachexia delayed rapid body weight loss. Thus, lack in NEP activity, genetically or pharmacologically, leads to a gain in body fat.

**Conclusions/Significance:**

In the present study, we have identified NEP to be a crucial player in the development of obesity. NEP-deficient mice start to become obese under a normocaloric diet in an age of 6–7 months and thus are an ideal model for the typical human late-onset obesity. Therefore, the described obesity model is an ideal tool for research on development, molecular mechanisms, diagnosis, and therapy of the pandemic obesity.

## Introduction

Human obesity is a very serious health disorder that has a polygenetic predisposition and is accompanied by dramatic consecutive morbidities (e.g. hypertension, enhanced risk of myocardial infarction, diabetes, and articular gout). In the last decades, overweight [BMI 25–30] and obesity [BMI beyond 30] has become an epidemic in industrial countries [1], [2]. According to UK statistics in 2006, 38% of adults were classified as overweight and 24% were classified as obese. Even 30% of children from the age of 2 to 15 were classified as overweight or obese. More than 5% of public health budgets in industrialized nations are spent on the treatment of obesity [3]. However, medical treatment of obesity to date has been disappointing with the greatest impact being bariatric surgery. Consequently, there is a critical need for new and innovative strategies to address the problem and molecular targeting is the focus of intensive biomedical research. Human obesity has a polygenetic causation [4]. In this context, genetically modified animals, especially several knockout mice are of greatest interest.

Recently, these knockout models have allowed the identification of several singular molecular components for the development of obesity [5], e.g. of leptin [6], insulin-(receptor) [7], interleukin-6 [8], GIP-(receptor) [9], pro-opiomelanocortin (POMC) and derivatives [10]. These publications substantially expanded the knowledge base on the molecular background of obesity. However, all of these studies described monogenetic reasons for the development of obesity. As mentioned above, the typical inherited human obesity has a polygenetic basis. Therapeutic approaches therefore need a target influencing multiple signaling pathways or functions, e.g. transcription factors, second messengers, or peptidases in order to reflect the polygenetic character of obesity. Among the peptidases the membrane-bound (type-II) metallo-enzyme neutral endopetidase (NEP or neprilysin, E.C.3.4.24.11) is of increasing interest as it generates or catabolises a large number of bioactive peptides and is widely distributed throughout the body (CNS, kidneys, lungs, testicles, blood vessels, etc.) [11], [12]. Promoted by the availability of specific inhibitors and of knockout mice, many biochemical and physiological properties of this peptidase have been described in detail. Typical substrates are the enkephalines, NPY, CCK, bradykinin, angiotensin, bombesin, ANP, the insulin chain, substance P [11], and the ”Alzheimer-peptide” β-Amyloid (Aβ) [13]. Importantly, among the NEP substrates is a couple of important orexigenic and anorexigenic compounds. The here presented results on the late-onset obesity are based on the availability of NEP-knockout mice; an animal model that plays also a crucial role in the discovery of the molecular background of further metabolic diseases [14].

## Results and Discussion

**Figure 1:**
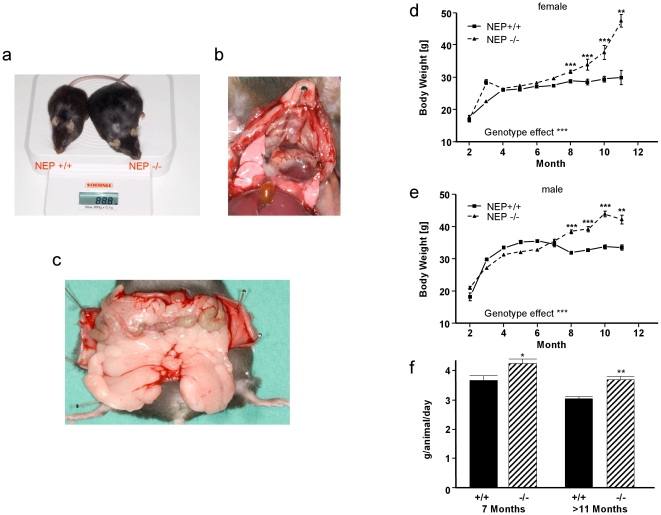
Age-related obesity in NEP-deficient mice. (**a**) Comparison of a NEP-knockout (NEP −/−) mouse with an age-matched wild-type animal (NEP +/+). (**b**) Steatosis of thorax and heart in a NEP-knockout mouse. (**c**) Abdominal fat accumulation in a NEP-deficient animal. Age-dependent development of body weight in (**d**) females and (**e**) males. Data is presented as means ± SEM. Where not shown, error bars lie within the dimensions of the symbols. Average per group 22 mice. Genotype effects ***P<0.001 by two-way ANOVA. Significant differences at specific times are calculated by Bonferroni *post-hoc* test, **P<0.01 and ***P<0.001. (**f**) Daily food consumption in 7 and >11 month-old female NEP-knockout (NEP −/−) mice compared with age-matched wild-type animal (NEP +/+), *P<0.05 and **P<0.01.

Using NEP-deficient mice and their wild-type controls for pathophysiological and biochemical analysis [15]–[17], we observed that mice with a genetic NEP deletion are characterized by a gain in body weight that became obvious only in elderly animals of both genders **(** **Figure 1a** **).** A first investigation of this effect identified a significant accumulation of fat tissue that was not restricted to abdominal fat **(** **Figure 1b and c** **)**. Detailed recording of body weight over one year identified significant weight differences for both, females and males, beginning in an age of approx. 7 months **(** **Figure 1d and e** **)** due to a continuation in body weight gain while wild-types reached a weight plateau. Extrapolated to the human, this time span in a mouse life corresponds to an age of approx. 35 to 40 years when many people become overweight. To investigate whether the gain in body weight was associated with increased food intake, we measured exemplarily in females the food intake at two different time points. When the differences between both genotypes first occurred (around the 7^th^ month) and in aged animals (>11 months), NEP-deficient mice ate significantly more than their age-matched wild-type controls (**Figure 1f**).

**Figure 2:**
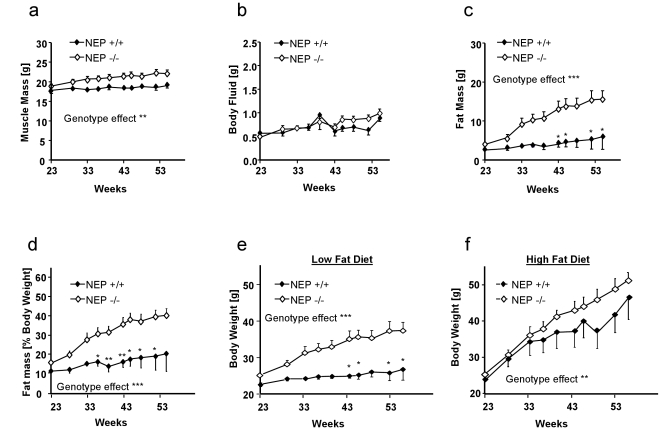
Body composition and diet-depending weight development in NEP-deficient mice. NMR-monitored influence of feeding on age-dependent body composition in mice fed with low fat diet divided in (**a**) muscle masses, (**b**) free body fluid, (**c**) fat masses, and (**d**) fat masses per body weight. Effects of (**e**) low fat and (**f**) high fat diet on the development of body mass. Data is presented as means ± SEM of at least 10 animals per group. Two-way ANOVA **P<0.01; ***P<0.001. Where not shown, error bars lie within the dimensions of the symbols. Genotype effects are calculated by two-way ANOVA (**P<0.01; ***P<0.001). Significant differences at specific time-points are calculated by Bonferroni *post-hoc* test, *P<0.05 and **P<0.01.

To further confirm that the weight gain in NEP-deficient mice is due to fat accumulation we used repeated *in vivo* NMR analyses during the time NEP-deficient mice developed their obese phenotype. Neither the amount of muscle mass nor of body fluid was altered in NEP knockouts (**Figures 2a and b**), whilst there was significant fat accumulation (**Figure 2c and d**). Notably, the increase in fat mass was comparable to the observed body weight gain. Investigations in both aged genotypes in a respiration chamber could not identify differences in their respiratory quotient (RQ-values wild-type: 0.743±0.006 versus knockout: 0.731±0.017) as well as in the emitted body temperature related energy (wild-type: 0.471±0.016 versus knockout: 0.476±0.049 kcal/h). Furthermore, the gain in body weight was also not associated with a lower locomotor activity as measured by radio-telemetry (data not shown).

To investigate whether the obese phenotype was modifiable by different food compositions, we fed females of both genotypes with either a low- or a high-fat diet (Mean food consumptions [in g/animal/d]; for low fat diet: wild-type: 3.3±0.4 versus knockout: 3.9±0.2; for high fat diet: wild-type: 3.0±0.1 versus knockout: 3.7±0.4). As shown in **Figure 2e**, the phenotype was much more pronounced under low fat diet, while high fat diet almost blunted the body weight differences due to a massive rise in body weight of wild-type mice **(** **Figure 2f** **)**.

**Figure 3:**
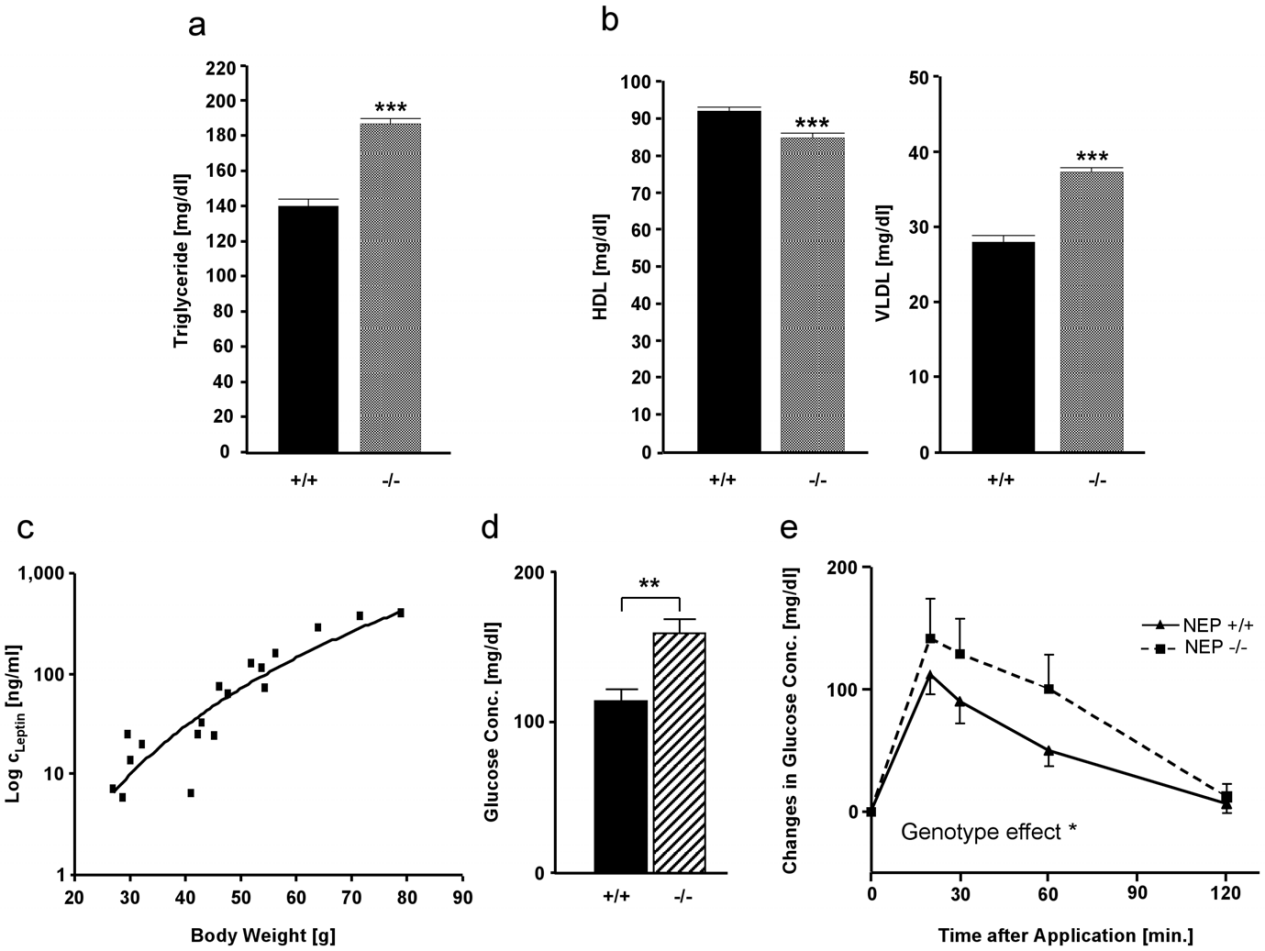
Biochemical parameters in obese NEP-deficient mice under low fat diet. (**a**) Serum triglycerides in one-year old NEP-deficient animals (−/−) and their age-matched wild-type controls (+/+). Student's *t*-test ***P<0.001 versus wild-type. (**b**) Serum HDL (left panel) and VLDL (right panel) in both genotypes. Data is presented as means ± SEM. Student's *t*-test ***P<0.001 versus wild-type. (**c**) Significant correlation of serum leptin levels in NEP-deficient mice with increasing body weight (r^2^ 0.92 [Pearson correlation]). (**d**) Comparison of basic glucose values (before glucose tolerance test) in plasma of NEP-knockout mice with wild-type animals. Student's *t*-test **P<0.01 versus wild-type. (**e**) Comparison of NEP-knockout mice (dotted line) with wild-type animals (solid line) in their response on a glucose tolerance test. Treatment differences are calculated by two-way ANOVA *P<0.05.

Obesity is often associated with a deregulation in non-complex lipid metabolism. As described for human obese people [18], our overweight NEP-deficient animals in an age of 12 months where characterized by higher concentration of serum triglycerides under normal food diet (**Figure 3a**) in comparison to their age-matched wild-type controls. Also fitting to the human situation, the analysis of cholesterol sub-fractions uncovered an unequal regulation. While the “good” HDL was significantly lowered (**Figure 3b, left panel**), the concentration of VLDL, a risk marker for cardiovascular diseases [18], [19], was significantly increased (**Figure 3b, right panel**).

**Figure 4:**
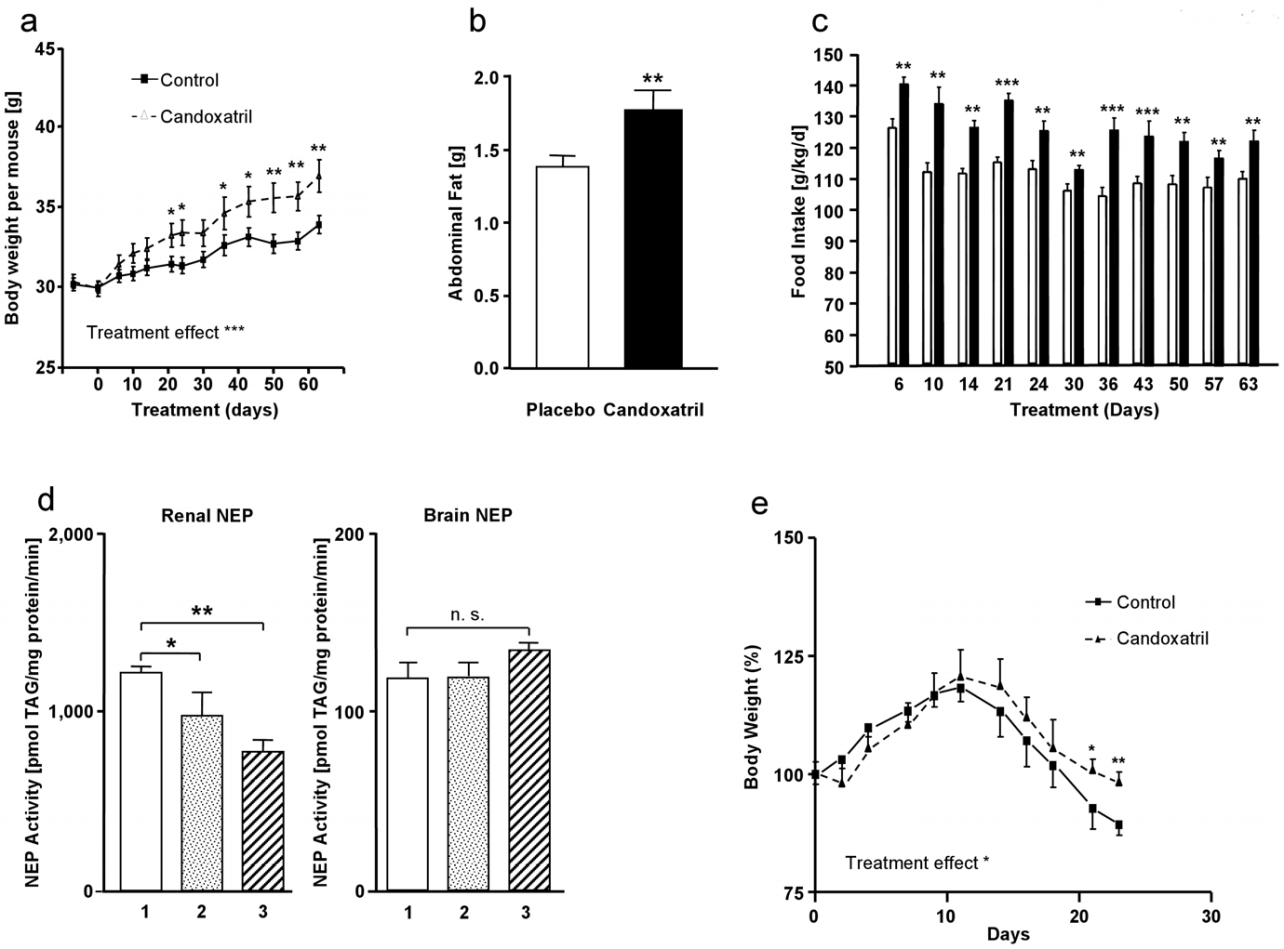
Effects of candoxatril (Pfizer, UK79,300) on body mass development. (**a**) Development of body weight in male C57BL/6 mice starting in an age of 6 months fed with standard diet, supplemented with placebo (solid line and squares) or of the NEP inhibitor candoxatril [consumption 200 mg/kg/day] (broken line and triangles). Treatment day 0 is the first day of treatment. Treatment effect ***P<0.001 by two-way ANOVA. Significant differences at specific time-points are calculated by Bonferroni *post-hoc* test, *P<0.05 and **P<0.01. (**b**) Abdominal fat in male C57BL/6 mice after feeding with standard food (open column) or with food supplemented with the NEP inhibitor candoxatril (black column) for two months [consumption 200 mg/kg/day]. Student's *t*-test **P<0.01 versus placebo. (**c**) Development of food intake in male C57BL/6 mice fed with standard food (open columns) or the same food supplemented with the NEP inhibitor candoxatril (black columns) [consumption 200 mg/kg/day]. Student's *t*-test **P<0.01, ***P<0.001 versus placebo. (**d**) Development of NEP activity in kidney (left panel) and brain (right panel) of mice after oral treatment with candoxatril (1) Placebo, (2) 100 mg/kg/day candoxatril, (3) 200 mg/kg/day candoxatril. Student's *t*-test *P<0.05, **P<0.01 versus placebo. (**e**) Development of body weight in male tumor-bearing mice (Pancreas carcinoma, PSN-1) fed with standard food (solid line and squares) or standard food supplemented with the NEP inhibitor candoxatril (200 mg/kg/day, broken line and triangles). Treatment effect *P<0.05, by two-way ANOVA. Significant differences at specific time-points are calculated by Bonferroni post-hoc test, *P<0.05 and **P<0.01.

Since it has been shown that leptin is a key molecule in the development of an obese phenotype [20], we investigated the role of this anorexigenic peptide hormone in the NEP knockout-mediated weight gain by measuring circulating leptin concentration in our animal model. As described for wild-type animals before, increasing leptin levels in NEP-deficient mice correlated with increasing body weight [9], [21] (**Figure 3c**). This normal regulation excludes a deficiency in leptin generation by fat tissue and therefore a reduced leptin-mediated control of food intake as being responsible for the fat accumulation in our NEP-deficient mice.

Since obesity is associated with a variety of secondary pathologies as diabetes mellitus type 2, we investigated whether obese NEP-deficient mice had alterations in glucose homeostasis and metabolism. Fasting blood glucose levels under basal conditions were significantly elevated in one-year old NEP-deficient mice (**Figure 3d**). Moreover, a glucose tolerance test revealed that these animals showed a significant weaker ability to handle an oral glucose load (**Figure 3e**). A similar trend between these genotypes has been described by Davidson *et al.* [14] for young animals.

The lack of compensation for the loss of NEP by functionally and structurally related peptidases (APN, ACE) in the untreated animals shows that NEP-deficient mice and their corresponding wild-type strain constitute an excellent animal model to characterize NEP-related processes [16], [22], [23]. However, a classical argument against data generated in knockout models is the modification of a variety of other gene products regulated secondarily to the life-long-gene deficiency, but being responsible for the described phenotype. Thus, we investigated, whether the obese phenotype in NEP-deficient mice could also be generated by a more acute pharmacological inhibition of that peptidase. There is data suggesting an effect of nonspecific NEP inhibition on food intake in sheep [24]; therefore we treated 6-month-old wild-type mice with the specific NEP inhibitor candoxatril (UK 79,300) given as a food additive [25]. Already after 20 days treatment, mice gained significantly more weight than sham-treated animals, and continued to do so over 40 days (**Figure 4a**). In accord with the results for the NEP-deficient mice due to genetic manipulation, the gain in weight mediated by candoxatril treatment was caused by a fat accumulation (**Figure 4b**). To answer the question whether the observed NEP-dependent fat accumulation is caused by stimulation of food intake or better food utilization, we recorded the daily food intake under candoxatril treatment. The data shown in **Figure 4c** clearly illustrate that the weight gain was caused by a significant increase in food consumption. Importantly, this orexigenic effect of candoxatril was already observed within the first measurements and thus supports the conclusion of NEP inhibition being primarily responsible for changes in peptide hormone concentrations that led to the observed stimulation in food intake.

In addition to its specificity, a second advantage of candoxatril is that it is thought not to pass through the blood brain barrier, thus discriminating between peripheral and central NEP effects on body weight. To check for candoxatril's ability to inhibit central NEP activity, we measured the peptidase's capacity to generate a NEP-specific degradation product in organs after application of different doses of candoxatril *in vivo* for 14 days. Neither a low nor a high dose of the NEP inhibitor had an effect on NEP activity in the brain cortex (**Figure 4d, right panel**). In contrast, peripheral NEP, as exemplarily shown for the kidney as one of the organs with highest NEP expression, was already significantly inhibited using the lower candoxatril concentration (**Figure 4d, left panel**).

All data shown here demonstrate a significant effect of lower NEP activity (either by pharmacological inhibition or genetic manipulation) on fat accumulation and thus pathological gain in body weight. However, there are pathophysiological circumstances such as tumor cachexia, where a rapid loss in weight would make stimulation in food intake a therapeutic option. The potential NEP inhibition of tumor-induced weight loss was investigated by use of candoxatril in nude mice bearing the human PSN-1 pancreatic carcinoma. This tumor type leads to a severe tumor cachexia very similar to the clinical situation. Tumor cachexia started 12 days after tumor implantation and achieved a maximum at the endpoint of the experiment, at day 23 after tumor inoculation (tumor weight loss >30% between day 12 and day 23). Treatment with candoxatril starting day 9 after tumor inoculation led to a significantly less pronounced loss in body weight compared with control-treated mice (**Figure 4e** **)**. The growth of the primary tumor and the weight of liver, spleen and kidney at day 23 after tumor implantation were not affected by NEP inhibition.

There is a critical need for novel treatment strategies for obesity, particularly as current oral medical treatment has had little impact. Molecular targeting may be one such approach. We have identified NEP to play a crucial role in food intake and in fat accumulation. Since the development of obesity in NEP-deficient mice starts at 6–7 months, this is an ideal model for typical human late-onset obesity. In contrast to the wide number of single genes described to be crucial in knockout animals to develop an obese phenotype, NEP fulfils the criteria for being a classical target in obesity with a polygenetic causation. This metallopeptidase hydrolyzes a large number of peptide hormones and is widely distributed throughout the body.

In contrast to other obesity models, our animals develop an obese phenotype under normocaloric conditions. Interestingly, with a high fat diet the significant difference between both genotypes declines, because the wild-type mice also become obese. This makes the NEP-deficient animals an even more relevant obesity model, since it reflects a classical situation where middle-aged people consuming food of normal (even healthy) quality become overweight whilst their diet remains unchanged. In addition, NEP-deficient animals develop lipid and glucose abnormalities, akin to type 2 diabetes, as the obesity develops [26].

A strong argument supporting NEP to be centered in food intake control is data of a reversal experiment. If reduction of NEP activity leads to stimulation in food intake, its pharmacological inhibition should prevent a massive reduction in body weight in a model of pathological body weight loss. We could demonstrate in mice bearing a pancreas carcinoma PSN-1 that specific NEP inhibition significantly reduces the loss in body weight until the experiment was stopped. Although the psychological impact in diseases like anorexia nervosa might be the dominant one, further experiments should evaluate whether the inhibition of NEP or signaling of NEP-targeted orexigens can reduce the body weight loss in diseases with pathological reduction in body weight.

As described above, NEP activity has massive impact on body mass and fat accumulation. The molecular background of this effect is hitherto not fully clarified, and a detailed description of the underlying mechanisms requires extensive research. On the other hand, on the basis of today's knowledge it might be predicted that the NEP-evoked degradation of substrates with pronounced orexigenic properties, such as NPY, galanin, or CNP should play an important role [27]–[29]. Own results and multiple references characterize these peptide hormones as proper substrates for mammalian NEP [11], [30], [31]. Thus, a deletion or a specific pharmacological inhibition of NEP may accumulate such orexigens and consequently lead to stimulated food intake and fat accumulation. The fact that the inhibition of peripheral NEP activity leads to the observed phenotype further reduces the number of candidate peptides. That the central NEP may not be important in appetite control makes the peptidase a promising pharmacological target, since it is not required that an inhibitor crosses the blood-brain barrier. This prevents alterations in complex central control mechanisms of behavior and emotional status. Over the last years, the model of a gut-brain axis has been developed [32]. That peripheral rather than central NEP is crucial for food intake and fat accumulation fits this model. NEP targets, which peripherally accumulate when NEP activity declines may signal to the brain and thus lead to an orexigenic stimulus.

Since the pharmacological increase in NEP might be difficult, the identification of orexigenic peptides (and their receptors) influenced by NEP activity may be a promising targeted approach to reduce body weight for the treatment of obesity.

## Materials and Methods

### Animals

We used male and female NEP-knockout mice that were originally generated by Lu *et al*. [33] and maintained in the breeding stocks of T.W. at the Charité, Campus Benjamin Franklin (CBF), Berlin, Germany. Experimental animals were bred from parents, which were F2 after hemizygous mating and being on a C57Bl/6N background. Animals were housed in litters separated according to sex at 22±1°C in a 12 h/12 h light/dark cycle with unrestricted access to food and water. Recordings of physiological parameters were performed between 9:00 a.m. and 1:00 p.m.

Experiments on adult mice were performed in accordance with the Guide for the Care and Use of Laboratory Animals published by the US National Institutes of Health (NIH Publication No. 85–23, revised 1996), the regulations of the Animal Care Committee of the Erasmus MC, and the Federal Law on the Use of Experimental Animals in Germany, and were approved by the local authorities (Landesamt für Gesundheit und Soziales des Landes Berlin).

### Diets

All animals obtained normal water al libitum and the following sorts of food:

Food I: Standard food; “ssniff SM/R/N-H (10 mm)”, (calorific value: 12.2 MJ/kg) ssniff Spezialdiäten GmbH, Soest, Germany, used for experiments described in Figure 1 and Figure 4.

Food II: (“Low fat diet”): “Altromin Standard-1324”; Altromin (calorific value: 12.5 MJ/kg; in this diet 13.6% of the convertible energy is attributed to the fat content), used for experiments described in Figure 2a–e.

Food III: (“High fat diet”): “Altromin C1057” (calorific value: 14.6 MJ/kg; in this diet 35.0% of the convertible energy is attributed to the fat content), used for experiments described in Figure 2f.

Food IV: Standard food (see above Food I) was cold milled, supplemented with the frequently used, well tolerated, orally active and tasteless NEP-inhibitor candoxatril (Pfizer) [34], [35], mixed and grouted under pressure (2 g/kg); this corresponds to a daily consumption of approx. 200 mg/kg/day. The manufacturing of candoxatril-containing food was performed as usually by the producer (ssniff/Germany). The used control food was prepared at the same day by the same management; used for experiments described in Figure 4.

### NMR analytics

Body composition was measured by NMR spectroscopy (Minispec MQ10 NMR Analyzer [Bruker, Billerica, Massachusetts/USA; www.minispec.com] in female mice using software by Echo Medical Systems [Houston, Texas, USA]).

### Respiration chamber and indirect calorymetry

Energy expenditure and metabolic fuel oxidation were estimated from oxygen consumption, carbon dioxide production, and urinary nitrogen excretion in open circuit metabolic cages as described previously by Aust *et al*. [36]. Oxygen concentration in the air was measured paramagnetically and carbon dioxide concentration by infrared absorption. From these values the respiratory quotient (RQ) was calculated. The parameters were controlled by the equipment of Braun & Hartmann (Minden, Germany) and stored online for each cage (mouse): Air flow; basic value for mice: 30 Nl/h performed by an air compressor; Actual O_2_ content (vol%); Actual CO_2_ production (ppm); Actual air pressure (hPa).

The indirect calorymetry was performed with single-housed mice in their home cages having a solid hermetically sealed lid.

### Biochemical analyses

Serum leptin levels were measured by ELISA (mouse leptin immunoassay kit Quantikine, MOB00, R&D Systems Inc., Minneapolis, USA). Lipids were quantified by the Cholestech LDX equipment (Cholesttech Corp., Hayward, CA, USA) according to the producer's manuals. All animals were in an age of 12 months.

### Glucose tolerance test

Eight female and 9 male wild-type as well as 10 female and 9 male NEP-knockout mice in an age of 12 months were tested for oral glucose tolerance. Basal blood glucose levels after 8 hour fasting were tested prior to the test (starting 8am). After oral application of a glucose solution (1 mg/g body weight) blood glucose levels were recorded after 30 min, 60 min, 90 min, and 120 min. The investigator was blinded to the genotypes.

### Measurement of NEP activity

NEP activity was measured according to Winkler *et al.* [37] using high performance liquid chromatography (HPLC) to monitor [D-Ala2, Leu5]enkephalin (DALEK, 100 µM) degradation and the parallel formation of Tyr–D-Ala–Gly in the presence of the aminopeptidase inhibitor bestatin (10^−4^ M) and the angiotensin-converting enzyme inhibitor lisinopril (10^−6^ M). The specificity of the reaction was characterised using 10^−5^ M candoxatrilat (Pfizer, UK73,967). Neutral endopeptidase activity was measured twice in all samples.

### Cachexia

Male NMRI:nu/nu mice aging 6 months (n = 16) were subcutaneously implanted with the human PSN-1 pancreatic carcinoma and equally allocated to a control (standard food) and treatment group (standard food + candoxatril; Food IV). Growth of the subcutaneous tumor and weight of the animals were daily monitored. Treatment was started on day 1 after tumor inoculation. Treatment was continuously performed until day 23 after tumor implantation (end of the experiment) whereby the researcher was blinded to the experimental groups. Weight of the liver, spleen, kidney and tumor was measured at the end of the experiment.

### Radio-telemetry experiment

Seven NEP-deficient mice and 7 age-matched wild-type controls received a telemetry implant (TA11-PA20; Data Sciences International, St Paul, Minnesota, USA) in the carotid artery as described earlier by Gross *et al*. [38]. In brief, the zero offset of the instrument was measured and the unit was soaked in 0.9% NaCl before implantation. Animals were anaesthetized with a mixture of ketamine (10 mg/kg) and xylasine (5 mg/kg). The transmitter catheter was inserted into the aortic arch via the left carotid artery. Thereafter, the catheter was sealed in place. After careful replacement of the intestine, the body of the transmitter was fixed on the abdominal wall and the cavity was closed with sutures. All mice were housed in individual cages in a sound-attenuated room. Beginning one week after surgery, the animals were monitored for 3 days receiving control diet.

### Data analyses

Data were analyzed by Student's t test or ANOVA with repeated measures, as appropriate.

Significant interactions identified by ANOVA were analyzed using a Bonferroni *post-hoc* test for pair wise comparisons. Statistical calculations were performed using Prism or Instat software from GraphPad, San Diego (USA). *P*<0.05 values were considered to be statistically significant.
